# Efficacy of N-acetylcysteine for Prevention of Postoperative Atrial Fibrillation Following Coronary Artery Bypass Grafting: A Systematic Review and Meta-Analysis of Randomized Controlled Trials

**DOI:** 10.31083/j.rcm2507243

**Published:** 2024-07-03

**Authors:** Dudy Arman Hanafy, Herick Alvenus Willim, Widya Trianita Suwatri, Alvin Ariyanto Sani, Hengky Khouw, Eunike Ita Susanti, Sugisman

**Affiliations:** ^1^Department of Cardiothoracic and Vascular Surgery, Division of Adult Cardiac Surgery, National Cardiovascular Center Harapan Kita - Faculty of Medicine, University of Indonesia, 10430 Jakarta, Indonesia

**Keywords:** N-acetylcysteine, postoperative atrial fibrillation, coronary artery bypass grafting, surgery

## Abstract

**Background:**

As the prevalence of coronary artery disease rises, the demand for coronary artery bypass grafting (CABG) increases. A common complication after CABG is postoperative atrial fibrillation (POAF), which is linked to adverse clinical outcomes. N-acetylcysteine (NAC), an antioxidant, may mitigate oxidative stress and reduce the incidence of POAF. This meta-analysis aims to investigate the efficacy of NAC in preventing POAF after CABG.

**Methods:**

The meta-analysis was conducted following Preferred Reporting Items for Systematic Reviews and Meta-Analyses (PRISMA) guidelines. We systematically searched multiple databases, including PubMed, Cochrane Library, ProQuest, and ScienceDirect, to identify relevant randomized controlled trials (RCTs). The intervention groups received perioperative NAC therapy, while the control groups received a placebo. The outcomes assessed were POAF incidence, all-cause mortality, and hospital length of stay (LOS). Review Manager 5.3 was used to conduct the meta-analysis.

**Results:**

Eleven RCTs involving 648 patients were included. The NAC group comprised 326 patients, while the control group comprised 322 patients. In the pooled analysis, patients in the NAC group had a significantly lower incidence of POAF (odds ratios (OR) = 0.57; 95% confidence intervals (CI) = 0.33 to 0.97; *p *= 0.04) and a shorter hospital LOS (weighted mean differences (WMD) = –0.66; 95% CI = –1.22 to –0.10; *p* = 0.02) compared to the control group. However, there was no significant difference in all-cause mortality.

**Conclusions:**

The perioperative administration of NAC can effectively reduce the incidence of POAF and hospital LOS in CABG patients. However, larger RCTs are needed to confirm these findings.

## 1. Introduction

Coronary artery disease (CAD) continues to be a leading cause of morbidity and mortality worldwide [1]. CAD was responsible for 20.5 million deaths in 2021, accounting for approximately one third of all deaths globally [2]. As the number of patients with CAD increases, so does the demand for coronary artery bypass grafting (CABG). CABG is a major surgical procedure involving the creation of new pathways to bypass blockages caused by atheromatous plaques in the coronary arteries [3]. It is indicated in cases of severe left main coronary artery disease and multivessel disease where percutaneous coronary intervention (PCI) would not be effective [4]. One aspect to consider in CABG patients is postoperative complications, which can negatively impact the patient’s survival and quality of life [5].

Postoperative atrial fibrillation (POAF) is one of the most common complications encountered after CABG. POAF is defined as the occurrence of new-onset atrial fibrillation (AF) in a patient with no prior history of AF that manifests within the first 4 weeks following surgery [6, 7]. The occurrence of POAF remains high, despite significant advancements in anesthesia and surgical techniques. The reported incidence of POAF after CABG varies between 20% and 40%, typically occurring between the second and fourth days following surgery, with the highest occurrence observed on the second day [8]. The incidence of POAF is reported to be similar regardless of the on-pump or off-pump CABG procedure [9]. POAF presents significant management challenges to clinicians as it is associated with unfavorable outcomes, including a deterioration in a patient’s hemodynamic status, an increased risk of heart failure, thromboembolic events such as stroke, prolonged hospital stays, and increased mortality [10].

Clinical investigations have revealed that patients who develop POAF after cardiac surgery exhibit elevated levels of inflammatory cytokines and oxidative stress damage compared to those who do not experience POAF. This suggests that an inflammatory response and oxidative stress may contribute to the development of POAF [11, 12]. Oxidative stress arises when there is a significant uncontrolled production of reactive oxygen species (ROS), surpassing the body’s natural antioxidant capabilities [13]. This phenomenon occurs during open-heart surgery that involves cardiopulmonary bypass (CPB) and cardioplegic arrest due to ischemia-reperfusion injury [13]. Oxidative stress has been demonstrated to induce a transient pro-arrhythmic substrate in the postoperative period and increase the risk of adverse outcomes [14]. Despite the well-known association, ROS-targeted therapy remains an often overlooked aspect in patients undergoing coronary revascularization procedures. Administering antioxidant agents in the perioperative period may decrease the release of ROS and mitigate oxidative stress [15].

N-acetylcysteine (NAC) is a compound derived from the amino acid cysteine and serves as a precursor for glutathione. NAC has antioxidant activities by scavenging free radicals and reducing oxidative stress. Due to this reason, its therapeutic promise extends to various diseases associated with oxidative stress, including cardiovascular diseases [16, 17]. Preliminary studies in animal models have shown that NAC can reduce ischemia-reperfusion injury and improve myocardial protection during CPB and cardioplegic arrest [18]. In a human study, the addition of NAC to blood cardioplegia in patients undergoing on-pump CABG can reduce myocardial oxidative stress [19]. Given that oxidative stress is a contributing factor to POAF, NAC may have a potential use in preventing POAF due to its antioxidant properties [17].

Prior meta-analysis studies have explored the effectiveness of NAC in preventing complications following cardiac surgery. However, the findings were inconsistent, and the populations studied included all types of cardiac surgeries, including valve and congenital heart surgery [20, 21, 22, 23]. To the best of current knowledge, no meta-analysis has specifically investigated the efficacy of NAC in preventing POAF in a specific population of CABG patients. Therefore, we conducted a systematic review and meta-analysis based on available randomized controlled trials (RCTs) to evaluate the efficacy of NAC in preventing POAF following CABG.

## 2. Methods

### 2.1 Study Registration

The study protocol for this research was registered in the International Prospective Register of Systematic Reviews (PROSPERO) under the registration number CRD42023469430.

### 2.2 Search Strategy

This meta-analysis followed the Preferred Reporting Items for Systematic Reviews and Meta-Analyses (PRISMA) guidelines [24]. A comprehensive systematic literature search was conducted across various databases, including PubMed, Cochrane Library, ProQuest, and ScienceDirect, to identify all published RCTs. The search utilized a combination of the following terms: (“N-acetylcysteine” OR “NAC”) AND (“postoperative atrial fibrillation” OR “POAF” OR “atrial fibrillation” OR “arrhythmia”) AND (“coronary artery bypass grafting” OR “CABG”). No restrictions were applied based on country, time, or language of publications. Additionally, the references cited in the relevant papers were manually examined to uncover any potential additional articles.

### 2.3 Inclusion and Exclusion Criteria

The inclusion criteria for eligible studies in this meta-analysis were as follows: (1) RCTs evaluating the effect of NAC in patients over 18 years old who had undergone on-pump or off-pump CABG; (2) NAC administered during the perioperative period; (3) RCTs that assessed the effect of NAC administration compared to a control group receiving a placebo; (4) Sufficient data available to compute the odds ratios (OR) for dichotomous variables or weighted mean differences (WMD) for continuous variables, along with their respective 95% confidence intervals (CI); (5) Studies available as full-text articles. The exclusion criteria were as follows: (1) Observational studies, review articles, case reports, letters, editorials, and conference abstracts; (2) Studies involving valve or congenital heart surgery; (3) Studies involving animal subjects; (4) Overlapping or duplicate studies.

### 2.4 Definition of Outcomes

The primary outcome of this meta-analysis was the incidence of POAF. The secondary outcomes were all-cause mortality and hospital hospital length of stay (LOS). POAF was defined as the occurrence of new-onset AF following CABG. All-cause mortality was defined as death resulting from any cause during hospitalization or within 30 days after surgery. Hospital LOS was defined as the number of days from hospital admission to discharge.

### 2.5 Data Extraction

Several pieces of information were collected from the selected articles, including the first author’s name, publication year, country of origin, baseline participant characteristics, previous medication, sample size, mean age, proportion of males and females, NAC protocol, incidence of POAF, all-cause mortality, and hospital LOS in each group.

### 2.6 Quality and Risk of Bias Assessment

The three authors (DAH, HAW, and WTS) independently assessed the quality of the included studies. In cases where discrepancies arose, additional discussions involving the remaining authors (AAS, HK, EIS, and S) were conducted to achieve a consensus. The Jadad score was employed as the quality assessment tool for the RCTs. A total score ranging from 1 to 5 points evaluating aspects such as randomization (0 to 2 points), blinding (0 to 2 points), withdrawals, and dropouts (0 to 1 point). Studies with a total Jadad score of 3 points or higher were categorized as high quality and low risk of bias, while those with scores of 2 points or lower were categorized as low quality and high risk of bias [25].

### 2.7 Statistical Analysis

Statistical analysis was performed using Review Manager 5.3 software (The Nordic Cochrane Centre, Copenhagen, Denmark). For dichotomous variables, we employed the OR, and for continuous variables, we used the WMD, both with 95% confidence intervals. The heterogeneity among the included studies was assessed using Cochran’s Q Chi-square test and the $I^{2}$ statistic. If there was no significant heterogeneity (*p* ≥ 0.05 and $I^{2}$ ≤ 50%), a fixed-effects model was applied. However, in cases of significant heterogeneity (*p* < 0.05 or $I^{2}$ > 50%), a random-effects model was applied. A *p*-value of less than 0.05 was considered indicative of statistical significance for all tests. The potential for publication bias was assessed by visually inspecting the funnel plot when the number of included studies for each outcome exceeded ten.

## 3. Results

### 3.1 Literature Search

**Fig. 1.**
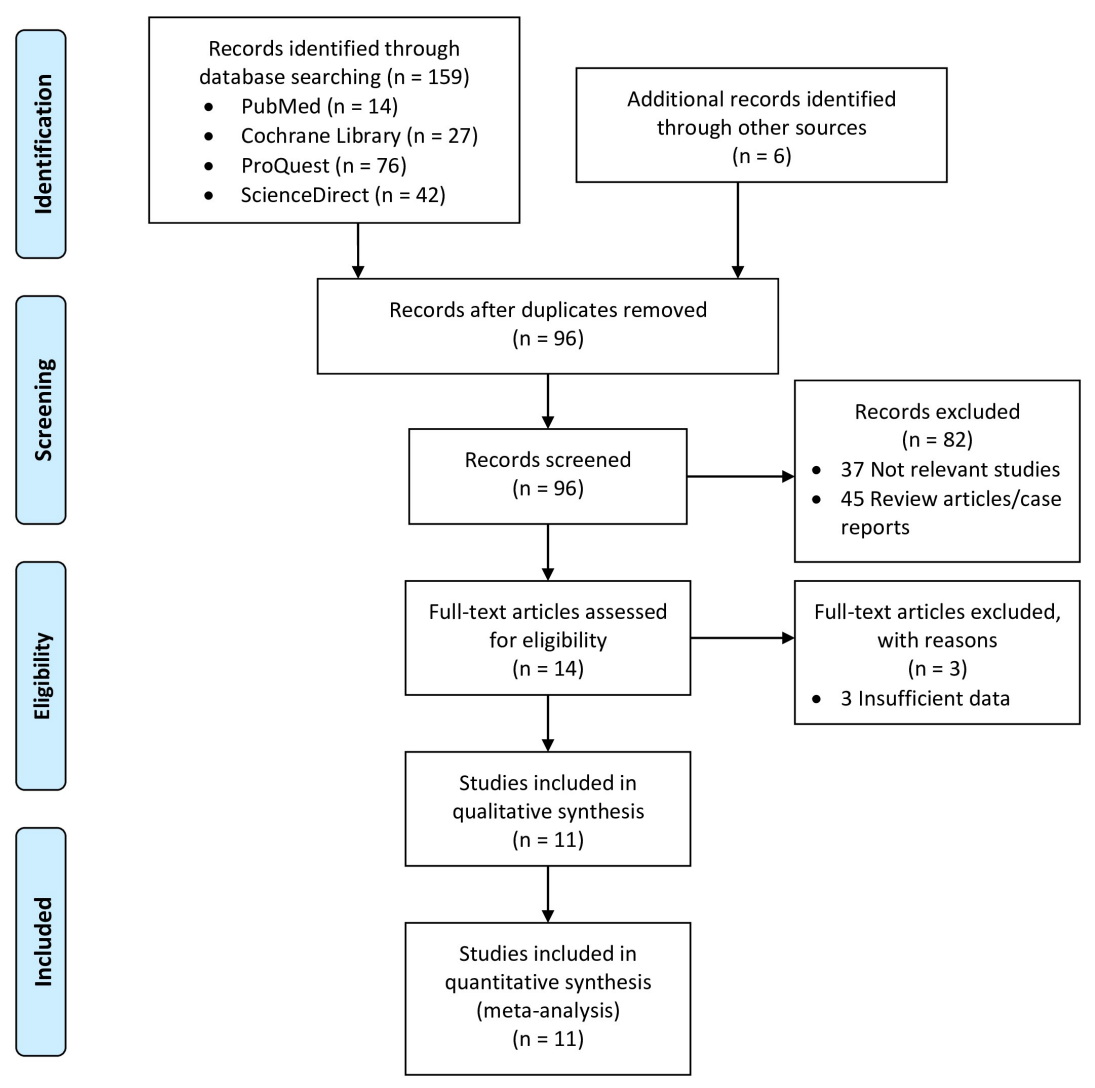
**Literature search flowchart**.

A systematic literature search was conducted across electronic databases, initially identifying 159 potential articles. This initial pool comprised 14 articles from PubMed, 27 articles from Cochrane Library, 76 articles from ProQuest, and 42 articles from ScienceDirect. Additionally, 6 articles were found through manual searches of relevant literature. After removing duplicates, 96 articles were screened based on titles and abstracts. Subsequently, 14 articles were chosen for full-text review, and 3 articles were excluded due to insufficient data. Finally, 11 articles were included in the meta-analysis. The flowchart illustrating this literature search process is presented in Fig. 1.

### 3.2 Study Characteristics and Quality Assessment

This meta-analysis included a total of 648 patients from 11 studies [26, 27, 28, 29, 30, 31, 32, 33, 34, 35, 36]. All studies were RCTs conducted between 2003 and 2018. The studies were conducted in various countries: one in Canada [26], six in Turkey [27, 28, 30, 31, 33, 35], one in Korea [29], one in Iran [32], one in Brazil [34], and one in India [36]. The sample sizes varied from 20 to 141 participants. The mean age of the NAC group ranged from 54 to 65 years old, and the mean age of the control group ranged from 53 to 65 years old. Of the 648 patients, 326 were in the NAC group, and 322 were in the control group. The characteristics of the included studies are presented in Table 1 (Ref. [26, 27, 28, 29, 30, 31, 32, 33, 34, 35, 36]). The NAC administration regimens differed among studies, with nine studies using the intravenous (IV) route [28, 29, 30, 31, 32, 33, 34, 35, 36], and two studies using the oral (PO) route before surgery, then continued by the IV route [26, 27]. The NAC regimen protocol of the included studies is presented in Table 2 (Ref. [26, 27, 28, 29, 30, 31, 32, 33, 34, 35, 36]). The Jadad score was used for quality assessment. All included studies have high-quality scores and low risk of bias. The Jadad scores of the included studies are presented in Table 3 (Ref. [26, 27, 28, 29, 30, 31, 32, 33, 34, 35, 36]).

**Table 1.** **Characteristics of the included studies**.

Reference	Year	Participant characteristics	Previous medication	N	Age (years)	Male (%)
El-Hamamsy *et al*. [26]	2007	Patients underwent CABG with CPB	BB, CCB, ACEI	Total: 100	NAC: 59.8 ± 7.8	NAC: 86%
				NAC: 50	C: 61.3 ± 7.4	C: 92%
				C: 50		
Erdil *et al*. [27]	2016	Patients underwent CABG with CPB	NR	Total: 82	NAC: 58.6 ± 10.1	NAC: 83%
				NAC: 42	C: 58.8 ± 9.9	C: 85%
				C: 40		
Eren *et al*. [28]	2003	Patients underwent CABG with CPB	NR	Total: 20	NAC: 61.1 ± 4.8	NAC: 80%
				NAC: 10	C: 60.5 ± 5.7	C: 70%
				C: 10		
Kim *et al*. [29]	2011	Patients with an LVEF <40% underwent off-pump CABG	BB, CCB, ACEI, ARB, diuretics	Total: 48	NAC: 60.8 ± 8.4	NAC: 87%
				NAC: 24	C: 65.3 ± 7.6	C: 92%
				C: 24		
Orhan *et al*. [30]	2006	Patients underwent CABG with CPB	NR	Total: 20	NAC: 59.6 ± 5.5	NAC: 70%
				NAC: 10	C: 61.8 ± 4.3	C: 60%
				C: 10		
Peker *et al*. [31]	2008	Patients underwent CABG with CPB	NR	Total: 40	NAC: 60.0 ± 11.4	NAC: 89%
				NAC: 19	C: 57.7 ± 8.6	C: 86%
				C: 21		
Soleimani *et al*. [32]	2018	Patients underwent CABG with CPB	NR	Total: 141	NAC: 62.4 ± 8.8	NAC: 54%
				NAC: 72	C: 60.7 ± 8.4	C: 49%
				C: 69		
Koramaz *et al*. [33]	2006	Patients underwent CABG with CPB	NR	Total: 30	NAC: 60.2 ± 1.8	NAC: 67%
				NAC: 15	C: 57.5 ± 2.1	C: 60%
				C: 15		
Santana-Santos *et al*. [34]	2014	Patients with CKD underwent off-pump or on-pump CABG	BB, CCB, ACEI, ARB, diuretics	Total: 70	NAC: 65.0 ± 8.2	NAC: 57%
				NAC: 35	C: 64.0 ± 9.0	C: 86%
				C: 35		
Karahan *et al*. [35]	2010	Patients underwent CABG with CPB	NR	Total: 44	NAC: 58.6 ± 2.7	NAC: 57%
				NAC: 21	C: 56.4 ± 3.1	C: 56%
				C: 23		
Prabhu *et al*. [36]	2009	Patients underwent CABG with CPB	NR	Total: 53	NAC: 54.2 ± 9.9	NAC: NR
				NAC: 28	C: 53.0 ± 8.1	C: NR
				C: 25		

ACEI, angiotensin-converting enzyme inhibitors; ARB, angiotensin receptor blockers, BB, beta-blockers; CCB, calcium channel blockers; CKD, chronic kidney disease; C, control groups; CABG, coronary artery bypass grafting; CPB, cardiopulmonary bypass; LVEF, left ventricular ejection fraction; NAC, N-acetylcysteine; NR, not reported.

**Table 2.** **NAC protocol of the included studies**.

Reference	NAC protocol
El-Hamamsy *et al*. [26]	600 mg PO the day before surgery and on the morning of surgery, 150 mg/kg IV before skin incision, then 12.5 mg/kg/h IV for 24 h
Erdil *et al*. [27]	600 mg/day PO for 3 days before surgery, then 300 mg via CPB prime solution
Eren *et al*. [28]	100 mg/kg IV 1 h before CPB, then 40 mg/kg/day for 24 h after CPB
Kim *et al*. [29]	100 mg/kg IV bolus over 15 min after anesthesia induction, then 40 mg/kg/day IV infusion for 24 h
Orhan *et al*. [30]	50 mg/kg IV at the start of anesthesia induction for 30 min
Peker *et al*. [31]	50 mg/kg IV 1 h before surgery, then 50 mg/kg/day IV for 48 h after surgery
Soleimani *et al*. [32]	50 mg/kg IV infusion over 30 min after anesthesia induction, then 2 × 50 mg/kg IV over 30 min for 48 h after surgery
Koramaz *et al*. [33]	50 mg/kg via the cold-blood cardioplegia
Santana-Santos *et al*. [34]	150 mg/kg IV over 2 h before surgery, then 50 mg/kg IV over 6 h after surgery
Karahan *et al*. [35]	50 mg/kg via the cold-blood cardioplegia
Prabhu *et al*. [36]	50 mg/kg via the isothermic cardioplegia

CPB, cardiopulmonary bypass; NAC, N-acetylcysteine; IV, intravenous; PO, oral.

**Table 3.** **Quality assessment of the included studies using the Jadad score**.

Reference	Randomization	Blinding	Withdrawals and dropouts	Total score
El-Hamamsy *et al*. [26]	1	2	0	3
Erdil *et al*. [27]	1	2	0	3
Eren *et al*. [28]	1	2	0	3
Kim *et al*. [29]	2	2	1	5
Orhan *et al*. [30]	1	1	1	3
Peker *et al*. [31]	2	1	1	4
Soleimani *et al*. [32]	2	2	1	5
Koramaz *et al*. [33]	2	1	0	3
Santana-Santos *et al*. [34]	2	2	0	4
Karahan *et al*. [35]	1	1	1	3
Prabhu *et al*. [36]	2	2	0	4

### 3.3 Effect of NAC on POAF

**Fig. 2.**
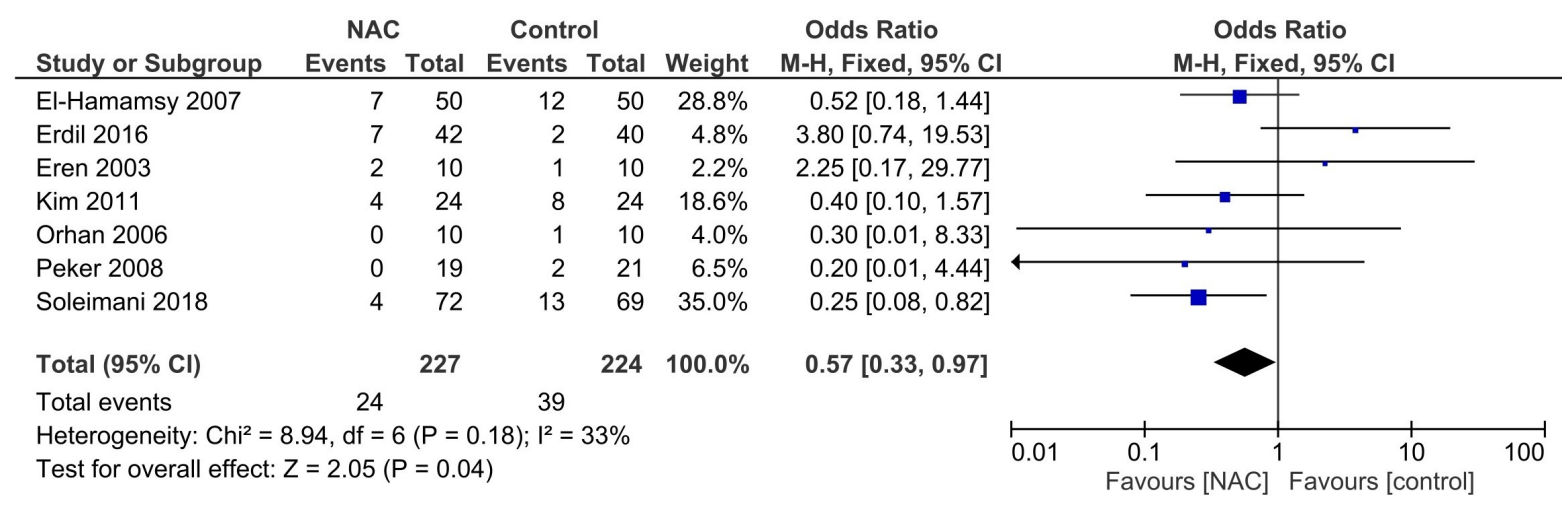
**Effect of NAC on postoperative atrial fibrillation. **NAC, N-acetylcysteine; M-H, Mantel-Haenszel.

Seven RCTs reported data on the incidence of POAF [26, 27, 28, 29, 30, 31, 32]. Details of the definition and assessment method of POAF are presented in Table 4 (Ref. [26, 27, 28, 29, 30, 31, 32, 33, 34, 35, 36]). The overall incidence of POAF was 14.0%. In the NAC group, POAF occurred in 10.6% (24 out of 227 patients), while in the control group, it occurred in 17.4% (39 out of 224 patients). No significant heterogeneity was found in the studies ($I^{2}$ = 33%; *p* = 0.18). The pooled analysis, using a fixed-effects model, revealed that NAC significantly reduced the incidence of POAF (OR = 0.57; 95% CI = 0.33 to 0.97; *p* = 0.04; Fig. 2) compared to the control group.

**Table 4.** **Definition and assessment method of POAF**.

Reference	Definition of POAF	Assessment method of POAF
El-Hamamsy *et al*. [26]	Any new-onset AF	NR
Erdil *et al*. [27]	Any new-onset AF	NR
Eren *et al*. [28]	Any new-onset AF	ECGs were performed on the first postoperative day
Kim *et al*. [29]	Any new-onset AF	NR
Orhan *et al*. [30]	Any new-onset AF	NR
Peker *et al*. [31]	Any new-onset AF	ECGs were performed continuously during the first 2 postoperative days, then twice daily when new symptoms occurred or rhythm abnormalities were detected on physical examination
Soleimani *et al*. [32]	Any new-onset arrhythmia that represents the characteristics of AF on the ECG lasting at least 30 s	ECGs were performed continuously during ICU and CCU stays
Koramaz *et al*. [33]	NR	NR
Santana-Santos *et al*. [34]	NR	NR
Karahan *et al*. [35]	NR	NR
Prabhu *et al*. [36]	NR	NR

AF, atrial fibrillation; ECG, electrocardiogram; NR, not reported; POAF, postoperative atrial fibrillation; ICU, intensive care unit; CCU, coronary care unit.

### 3.4 Effect of NAC on All-Cause Mortality

**Fig. 3.**
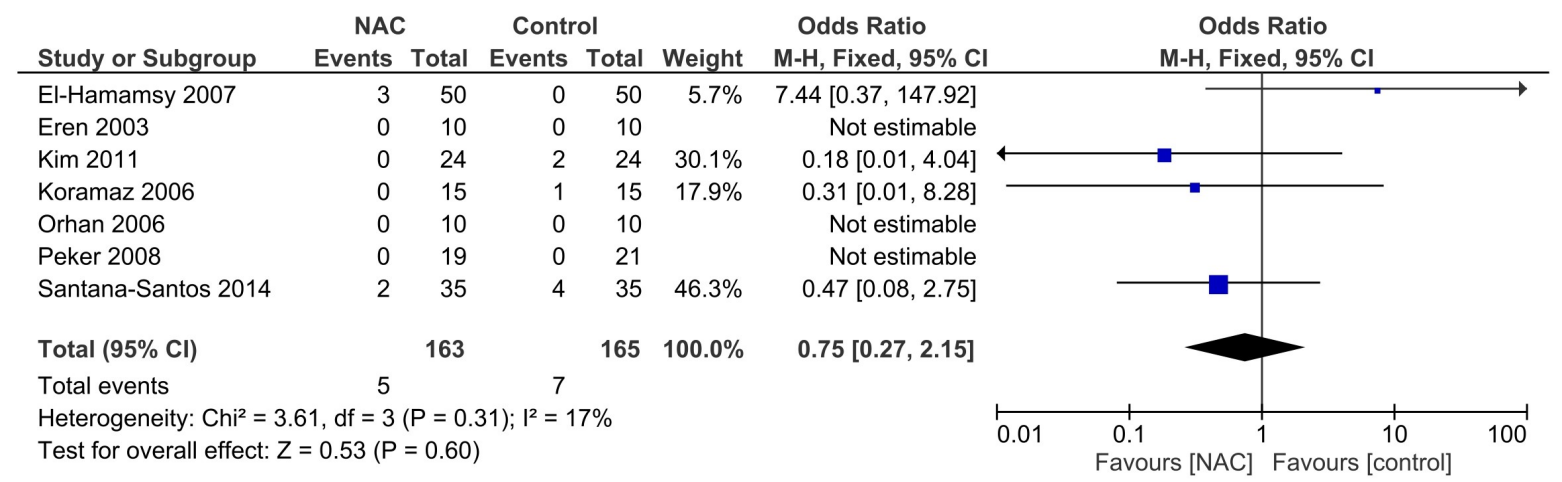
**Effect of NAC on all-cause mortality. **NAC, N-acetylcysteine; M-H, Mantel-Haenszel.

Seven RCTs reported data on all-cause mortality [26, 28, 29, 30, 31, 33, 34]. No significant heterogeneity was found in the studies ($I^{2}$ = 17%; *p* = 0.31). The pooled analysis using a fixed-effects model revealed that NAC did not significantly reduce all-cause mortality (OR = 0.75; 95% CI = 0.27 to 2.15; *p* = 0.60; Fig. 3) compared to the control group.

### 3.5 Effect of NAC on Hospital LOS

**Fig. 4.**
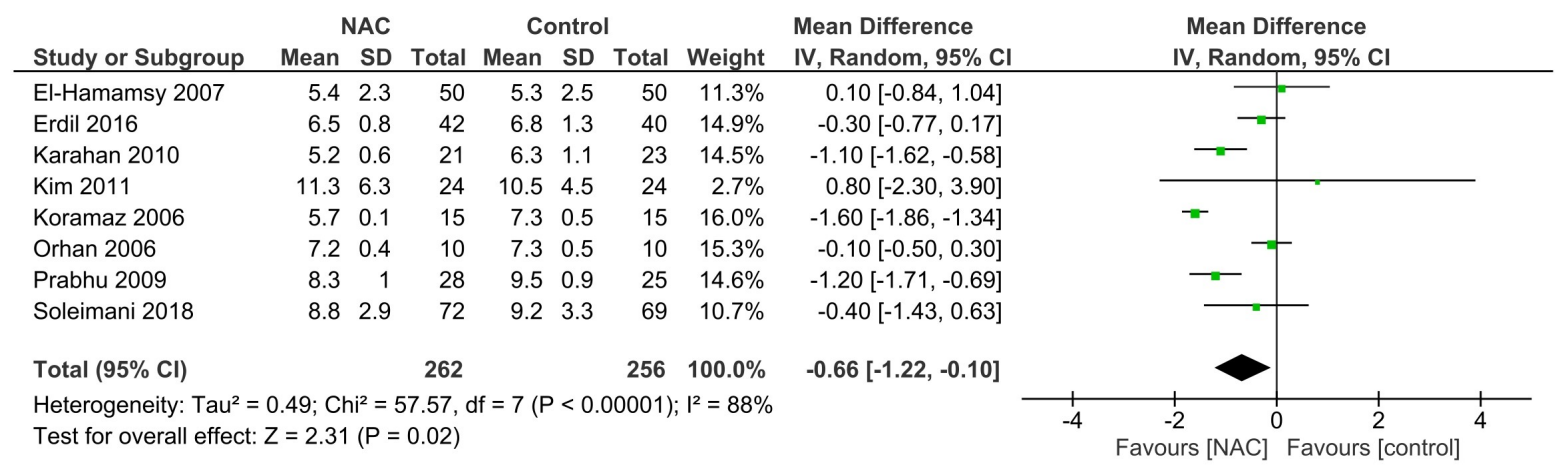
**Effect of NAC on hospital length of stay. **NAC, N-acetylcysteine; IV, inverse variance.

Eight RCTs provided data on hospital LOS [26, 27, 29, 30, 32, 33, 35, 36]. Significant heterogeneity was found in the studies ($I^{2}$ = 88%; *p* < 0.00001). The pooled analysis, utilizing a random-effects model, indicated that NAC significantly reduced hospital LOS (WMD = –0.66; 95% CI = –1.22 to –0.10; *p* = 0.02; Fig. 4) compared to the control group.

### 3.6 Publication Bias

A funnel plot cannot be generated in our meta-analysis due to the limited number of included RCTs for all outcomes.

## 4. Discussion

This systematic review and meta-analysis incorporated eleven RCTs, involving a total of 648 patients undergoing CABG. The pooled analysis revealed a significant association between perioperative NAC therapy and a halved risk of POAF compared to those in the control group. Additionally, the NAC group demonstrated a shorter hospital LOS compared to the control group. These findings provide insights into the potential of NAC in mitigating POAF, suggesting that NAC may play a valuable role in enhancing outcomes following CABG by preventing the occurrence of POAF. To the best of current knowledge, this is the first meta-analysis studying the efficacy of NAC for the prevention of POAF, specifically in the isolated CABG population. Previous meta-analyses by Pereira *et al*. [22] and Wang *et al*. [23] found that perioperative NAC supplementation did not reduce the incidence of POAF. However, these studies included all RCTs involving various types of cardiac surgeries, including valve surgeries [22, 23]. In our meta-analysis, only CABG patients were included.

Risk factors for POAF include older age, preoperative anemia, a history of hypertension, myocardial infarction, renal failure, heart failure, and chronic obstructive pulmonary disease (COPD). Additional risk factors include reduced left ventricular ejection fraction, the type of operation (valve surgeries increase the risk), a longer perfusion time, the use of intra-aortic balloon pump, and the use of inotropes [37, 38]. Inflammation responses following surgery, both at the systemic and local levels, can result in oxidative injury by releasing ROS. ROS can trigger adverse changes in myocardial electrical activity, including a shortened effective refractory period in the action potential, which can increase the likelihood of developing POAF [39]. Considerable experimental evidence supports the occurrence of oxidative injury in the myocardial tissues of patients with AF [40]. Moreover, studies have indicated elevated levels of serum markers of myocardial oxidation, such as superoxide and peroxynitrite, in patients who developed POAF [40, 41, 42]. Atrial nicotinamide adenine dinucleotide phosphate (NADPH) oxidase activity, which serves as the primary source of superoxide in the atria, was higher in patients who developed POAF compared to patients who maintained sinus rhythm [43]. In light of these experimental findings, perioperative antioxidant supplementation may play a role in reducing the incidence of POAF.

NAC serves as an antioxidant known for its ability to alleviate cellular oxidative damage. Studies indicate that NAC can diminish reperfusion-related arrhythmias, ischemia-reperfusion injury, and restrict the expansion of infarcted areas [44, 45]. When employed alongside reperfusion therapy in acute myocardial infarction patients, NAC has shown promise in reducing oxidative stress and preserving left ventricular function [46]. Furthermore, NAC has demonstrated positive outcomes in COPD, acknowledged as another risk factor for in development of POAF [47, 48]. NAC acts as a precursor to glutathione, enhancing its synthesis by entering cells and converting it into cysteine. The stimulation of glutathione production leads to elevated levels of intracellular reduced glutathione, often depleted in response to increased inflammation and oxidative stress [49]. Additionally, NAC may inhibit the renin-angiotensin system and alleviate atrial remodeling through its antioxidant and anti-inflammatory properties [50]. Since inflammation and oxidative stress contribute to the development of POAF, NAC holds promise as an agent for reducing its incidence. Other antioxidants reported to potentially decrease the occurrence of POAF include polyunsaturated fatty acids, vitamin C, and vitamin E [51, 52].

The protocol for administering NAC varies across studies, encompassing dosage (ranging from 40 to 150 mg/kg/day), administration routes (intravenous, oral, oral plus intravenous, or as an addition to cardioplegia), and duration (1 hour to 2 days). Currently, the standard dose and optimal duration of NAC administration remain unclear. NAC can be reasonably administered up to 2–3 days postoperatively. Previous research has suggested that inflammatory cytokines peak on days 2–3 post-operation, aligning with the day of the highest incidence of POAF [53, 54]. The diversity in NAC administration protocols emphasizes the imperative for additional research to establish standardized guidelines for its application in CABG. A comprehensive understanding of the ideal dose and duration could pave the way for more consistent and effective prevention of POAF, ultimately enhancing patient outcomes and facilitating recovery.

We observed no significant difference in all-cause mortality between the NAC group and the control group. This result aligns with a previous meta-analysis conducted by Zhao *et al*. [20], which also reported no association between NAC and all-cause mortality. Interestingly, our meta-analysis revealed that NAC supplementation may reduce hospital LOS. In a prior meta-analysis by Ali-Hassan-Sayegh *et al*. [55], NAC did not exhibit a significant reduction in hospital LOS following cardiac surgery. It is worth noting that their study encompassed all types of cardiac surgeries, including valve surgeries, whereas this meta-analysis specifically focused on CABG [55]. Tamis and Steinberg *et al*. [56] highlighted that POAF after CABG was independently linked to prolonged hospitalization. They found that patients developing POAF had a hospital LOS 3.2 days longer than those who did not experience POAF [56]. One plausible explanation for the observed reduction in hospital LOS with NAC supplementation could be its potential to decrease the incidence of POAF. Hospital LOS stands out as a crucial outcome in the context of CABG patients. Studies indicate that an extended hospital stay correlates with heightened postoperative complications, increased financial burdens, and diminished functional capacity levels, all contributing to a reduction in overall quality of life [57, 58].

This meta-analysis has several limitations. The total number of patients included in this meta-analysis was relatively small, potentially limiting the generalizability of the findings to a global population. Furthermore, there was no standardized protocol regarding the dosage, route of administration, and duration of NAC used. The funnel plot analysis could not be performed due to the limited number of included studies, meaning the possibility of publication bias cannot be ruled out. To address these limitations, future research should prioritize conducting larger, well-designed RCTs to confirm these findings and provide more precise estimates of the efficacy and safety of NAC in preventing POAF after CABG.

## 5. Conclusions

The results of this systematic review and meta-analysis provide evidence that perioperative NAC administration is associated with a lower incidence of POAF and shorter hospital LOS in patients undergoing CABG. These findings highlight the potential benefits of NAC in improving outcomes following CABG. However, larger RCTs in the future are needed to further enhance understanding of the efficacy and safety of NAC and to establish the optimal dosage.

## Data Availability

The data used to support the findings of this study are included within the article.

## Supplementary Material

Supplementary material associated with this article can be found, in the online version, at https://doi.org/10.31083/j.rcm2507243.
